# Microglia activated by microbial neuraminidase contributes to ependymal cell death

**DOI:** 10.1186/s12987-021-00249-0

**Published:** 2021-03-23

**Authors:** María del Mar Fernández-Arjona, Ana León-Rodríguez, María Dolores López-Ávalos, Jesús M. Grondona

**Affiliations:** Laboratorio de Fisiología Animal, Departamento de Biología Celular, Genética y Fisiología, Facultad de Ciencias, Universidad de Málaga, Instituto de Investigación Biomédica de Málaga-IBIMA, Campus de Teatinos, 29071 Málaga, Spain

**Keywords:** Microglia, Ependyma, Neuraminidase, Sialic acid, Neuroinflammation, Interleukin-1β, Rats

## Abstract

The administration of microbial neuraminidase into the brain ventricular cavities of rodents represents a model of acute aseptic neuroinflammation. Ependymal cell death and hydrocephalus are unique features of this model. Here we demonstrate that activated microglia participates in ependymal cell death. Co-cultures of pure microglia with ependymal cells (both obtained from rats) were performed, and neuraminidase or lipopolysaccharide were used to activate microglia. Ependymal cell viability was unaltered in the absence of microglia or inflammatory stimulus (neuraminidase or lipopolysaccharide). The constitutive expression by ependymal cells of receptors for cytokines released by activated microglia, such as IL-1β, was demonstrated by qPCR. Besides, neuraminidase induced the overexpression of both receptors in ventricular wall explants. Finally, ependymal viability was evaluated in the presence of functional blocking antibodies against IL-1β and TNFα. In the co-culture setting, an IL-1β blocking antibody prevented ependymal cell death, while TNFα antibody did not. These results suggest that activated microglia are involved in the ependymal damage that occurs after the administration of neuraminidase in the ventricular cavities, and points to IL-1β as possible mediator of such effect. The relevance of these results lies in the fact that brain infections caused by neuraminidase-bearing pathogens are frequently associated to ependymal death and hydrocephalus.

## Introduction

Ependymal cells form a cuboidal monostratified epithelium that covers the wall of the cerebral ventricles and the central canal of the spinal cord. One of their distinctive morphological characteristics is that they are multiciliated, which allows them to perform an asymmetric shake that generates a constant flow of cerebrospinal fluid (CSF) [1, 2]. Ependymal cells are linked by different types of cellular junctions, like cadherins and integrins [3–5], that maintains the integrity of the ependymal epithelium. Also, the use of sialic acid-specific lectins established that the luminal surface of the ependyma is rich in sialic acid, a feature that might be relevant to various properties such as epithelial permeability and integrity, cell masking in innate immunity, or CSF dynamics [6–8]. The integrity of the ependymal epithelium is essential for the stability of the ventricular system [7, 8] and it has been shown that the absence of ependyma [9, 10] or a lack of ciliary beating [1, 11–15] can cause hydrocephalus.

Neuraminidase (NA) is a sialidase found in the membranes or cell walls of certain viruses and bacteria. Some of these microorganisms displaying NA in their coats induce damage in ependymal cells when invading the central nervous system (CNS), as occurs with *Streptococcus pneumoniae*. This bacteria, which is the main cause of bacterial meningitis in humans [16], also provokes ependymal damage [17, 18]. In addition, NA-bearing viruses from orthomyxoviridae family (influenza viruses), and paramyxoviridae family (causing mumps and measles) have been associated to ependymal death and hydrocephalus [19–28].

In rats, a single intracerebroventricular (ICV) injection of NA into the lateral ventricles induces an acute sterile neuroinflammation, which disappears about 2 weeks later [6]. This neuroinflammation model is characterized by a strong activation of microglia and astrocytes in areas near the ventricles, and infiltration of neutrophils, monocytes, CD8 + T lymphocytes, and B lymphocytes from the bloodstream [6]. In addition, a high dose of NA (Cat. nº 107,590, Boheringer, Mannheim GmbH, Biochemica, Germany) causes a massive destruction of the ependymal epithelium lining the ventricular walls, occlusion of the cerebral aqueduct and the development of hydrocephalus [7]. With a lower dose of NA (Cat. nº 1 585 886, Roche Diagnostics GmbH, Germany), only limited patches of the ependyma are lost (53.5 ± 8.5%)[29]. However, this loss is permanent since the ependyma does not regenerate; a glial scar appears in the nude surfaces [30].

Recently, the activation of the complement system by NA has been demonstrated, as well as its participation in ependymal cell death; however, complement alone does not account for ependymal death, since some damage occurred even when the complement was not active [29]. Due to the relevance of the ependyma in the stability of the ventricular system [7, 8] and in preventing hydrocephalus [9, 31–33], investigating the causes of the ependymal loss provoked by microbial NA is of outstanding interest. As activation of microglia in periventricular areas is an outstanding feature of NA-induced inflammation, we hypothesize that activated microglia might participate in ependymal cell death. Recently, the direct activation of microglia by NA acting through toll-like receptor 4 (TLR4) has been demonstrated [34].

Explants obtained from ventricular walls represent a valuable and complex culture model where tissue cytoarchitecture is preserved. A layer of subependymal microglia lies underneath the ependymal epithelium [35]. To assess the effect of NA-activated microglia on ependymal cells, the viability of ependymal cells in ventricular wall explants exposed to NA in vitro was quantified. To further investigate the impact of NA-activated microglia on ependymocytes, pure cultures of isolated ependymal cells were co-cultured with isolated microglial cells in the presence of NA. A possible mechanism of ependymal death involving pro-inflammatory cytokines was later inquired. Therefore, this work aimed to explore the contribution of NA-activated microglia to the impaired viability of ependymocytes, and the possible role of specific pro-inflammatory cytokines.

## Material and methods

### Animals

Male Wistar rats (9 weeks old; 350 g weight) were provided by Charles River Laboratories (Barcelona, Spain). These animals were maintained in the animal house at Universidad de Malaga, under a 12 h light/dark cycle, at 23 °C and 60% humidity, with food and water available ad libitum. To obtain primary cell cultures, rats were anaesthetized with 2,2,2-tribromoethanol (300 mg/kg i.p.) before decapitation. Animal care and handling was performed according to guidelines established by Spanish legislation (RD 53/2013) and the European Union regulation (2010/63/EU). All procedures were approved by the ethics committee of Universidad de Malaga (Comite Etico de Experimentacion de la Universidad de Malaga; reference 2012–0013). All efforts were made to minimize the number of animals used and their suffering.

### Ventricular wall explants

Explants were obtained from the wall of the lateral ventricles of adult rats, following the procedure described by Grondona et al. [36]. They were about 0.5 mm thick and included about 1.0–1.5 mm^2^ of ventricular surface. These explants were used for: (i) obtaining pure cultures of ependymal cells, (ii) experiments of co-culture with microglial cells, (iii) cytokine receptor gene expression studies.

### Pure cultures of ependymal cells

Pure ependymal cell cultures were obtained from ventricular wall explants as described by Grondona et al. [36]. Briefly, the explants were initially washed with ice-cold HBSS without calcium and magnesium (Invitrogen, ref. 14170-088) for 30 min, and then incubated with TrypLE™ Express (Invitrogen, ref. 12605-010) following a specific sequence of temperatures: 4 °C [5 min], 37 °C (20 min), and 4 °C (5 min). The explants were then washed for 10 min in ice-cold alpha-MEM (Invitrogen, ref. A10490-01) with the following additives: 0.2% Pluronic F-127 (Sigma, ref. P-2443), 0.3% glucose (Sigma, ref. D-7021), and 0.01 M HEPES (Invitrogen, ref. 15,630,056). Finally, they were incubated at 37 °C and 5% CO_2_ during 24 h in a separation medium consisting of αMEM supplemented with 0.2% Pluronic F-127, 0.3% D-glucose, 0.01% DNase type I (Sigma, ref. DN-25) and 0.01 M HEPES. After 24 h, detached ependymal cells, which were free floating and moving in the medium, were harvested by centrifugation (300*g*, 10 min) and suspended in the supplemented αMEM (described above). Isolated ependymal cells were used for viability studies in co-culture with microglial cells, as well as to determine the expression of specific cytokine receptors. For co-culture experiments, the ratio microglial cells/ ependymal cells was about 3.3. This ratio was estimated considering (i) the relative proportion of both cell types in vivo in the rat lateral ventricles (ratio ≈0.24), (ii) the intimate relationship of ependymal cells to subependymal microglia (which is lost in the co-culture system), and (iii) the relatively high dilution of microglial secretion within the culture media (a volume much larger than that of the ventricular cavities).

### Isolation and culture of microglial cells

Microglial cells were isolated according to the Saura´s method [37]. The mix cells cultures were obtained from of 3 to 5-day-old rats sacrificed by decapitation. The purity of these microglial cultures was checked by immunocytochemistry and was about 95%. The average yield with this method was about 20,000 cells/well in 12 multiwell plates (Sigma-Aldrich, TPP tissue culture plates, Z707775). Microglial cells survived in culture for at least 2 weeks (co-culture experiments were performed at shorter times) in the presence of conditioned media obtained from the previous mixed cultures. Rat microglial cultures were used for co-culture experiments with either explants or ependymocytes.

### Viability assay for ependymal cells in explants

Lateral ventricular wall explants (septal and striatal sites) obtained from adult rats were used to assess ependymal cell viability under different experimental conditions. Explants were individually placed in 12-well plates in DMEM-F12 medium, supplemented with 10% FBS and 1% penicillin/streptomycin (1 ml per well). The explants were co-cultured with pure primary microglial cells (20,000 microglial cells per well). Four independent experiments with explants and microglial cultures obtained from different rats were performed.

Microglia activation was achieved by the addition to the culture medium of lipopolysaccharide (LPS; InvivoGen, LPS-EB Ultrapure E. coli 0111:B4; 1 μg/mL) [38, 39] or NA (Neuraminidase, Roche, *C. perfringens* 11 585 886 001; 50 mU/mL) [40]. Other conditions consisted of: (i) explants treated with NA without microglia, and (ii) explants co-cultured with non-activated microglia. All these culture conditions were maintained for 24 h. Then, the viability assay was performed as follows. Explants were incubated for 10 min in a 0.4% solution of the vital stain trypan blue (Gibco; 15250061). After staining they were washed with HBSS for 2 min, immersed in Bouin´s fixative solution for 2 h (5% acetic acid, 9% formaldehyde, and 0.9% picric acid), and later embedded in paraffin wax. Five-micrometer paraffin sections were obtained from each explant, aiming to get a cutting plane perpendicular to the ependymal surface, so that ependymal cells could be clearly identifiable. Paraffin sections were mounted onto slides treated with poly-l-lysine solution (Sigma-Aldrich; P8920). After deparaffinization, tissue sections were stained with hematoxylin to visualize the tissue and to stain live cells, while dead cells were distinguished by a blue staining (Fig. 2). Images were captured using an Olympus VS120 microscope through UPLSAPO 20 × objective. About 400 live (white) or dead (blue) ependymal cells were counted per explant; viability was expressed as the percentage of living cells.

### Viability assay for cultured ependymal cells

Primary cultures of ependymal cells obtained from adult rats were placed in DMEM-F12 medium supplemented with 10% FBS and 1% penicillin/streptomycin. Cells were seeded at a density of 1500 cells/well in a 24-multiwell plate, using 0.5 ml of media per well. Some ependymal cells were placed in wells already containing a pure microglial culture (from rat) previously prepared. To trigger microglial activation, LPS or NA were added (same concentrations as described for explants); some wells were left without NA or LPS as controls. LPS or NA were also added to wells with ependymal cells but with no microglia. Incubation times were 1, 3, 6 and 24 h; six independent experiments with ependymal and microglial cultures from different rats were done. After incubation under the different experimental conditions, ependymal cells were immediately processed to evaluate their viability by treating them with a 0.4% trypan blue solution for 5 min. Then, they were centrifuged and the pellet fixed with 4% paraformaldehyde (PFA) (Sigma-Aldrich; ref. 1.00496) for 5 min, and washed with 0.9% sterile saline. Live (white) and dead (blue) cells were counted under the microscope by using a Neubauer chamber. Cells were counted following a standardized path along the Neubauer chamber, until reaching about 400 cells per sample, which represent approximately one third of the ependymal cells seeded in each well. All counting were performed blinded for experimental groups. Viability was expressed as percentage of living cells.

For experiments with functional blocking antibodies to cytokines anti-IL-1β (rat IL-1β/IL-F2 Antibody, R.D. Systems, AF-501-NA) and anti-TNFα (rat TNFα Antibody, R. D. Systems, AF-510-NA) were added to the culture medium at 5 μg/mL and 2.5 μg/mL respectively; ependymocytes viability was determined 24 h later. Eight independent experiments with ependymal and microglial cultures obtained from different rats were performed.

### Immunocytochemistry

Ependymal and microglial cell cultures were fixed in 4% PFA for 10 min. The primary antibodies used were as follows: rabbit anti-IBA1 (1:1000, WAKO, 19-19741); goat anti-IL-1β (1:500, R&D Systems, AF501NA); and mouse anti-β-IV tubulin (1:1000, monoclonal Sigma T7941). The secondary antibodies used were as follows: biotinylated goat anti-mouse IgG (H + L) (1:1,000, Thermo Fischer Scientific 31800), donkey anti-rabbit Alexa 488 (1:1000, Thermo Fischer Scientific A-21206); and donkey anti-goat Alexa 594 (1:1000, Thermo Fischer Scientific A-11058). Both immunoperoxidase and immunofluorescence labelling was performed as described elsewhere [34, 40]. Negative controls for the immunostaining consisted in equivalent cultures subjected to the same protocol but omitting the primary antibody.

### Specificity of the primary antibodies

The specificity of anti-IBA1 (WAKO, 19-19741) (immunogen: synthetic peptide corresponding to C-terminus of Iba1) and mouse anti-β-IV tubulin (Sigma T7941, monoclonal antibody produced by the ONS.1A6 hybridoma; immunogen: synthetic peptide from the C-terminal sequence of β-tubulin isotype IV coupled to BSA) were validated by the manufacturer, as given in the specification sheets.

### Gene expression by quantitative PCR

Total RNA from ependymal cells, choroid plexus (obtained from the lateral ventricles) and brain parenchyma tissue (the latter obtained from striatal wall) was isolated using TRIzol reagent (Invitrogen; ref. 15596026), following manufacturer´s instructions. The concentration of RNA was measured in a NanoDrop microvolume spectrophotometer (NanoDrop 1000, Thermo Fisher Scientific). The A260/280 ratio of the isolated RNA was usually about 1.8. cDNA synthesis from isolated RNA was performed using the SuperScript TM III First-Strand Synthesis (Invitrogen; 11752-050) according to the manufacturer´s protocol.

To quantify specific messenger ribonucleic acids (mRNAs) (Table 1) in the cDNA samples, the SYBR Green I based method for qPCR was employed. The hot start reaction mix FastStart Essential DNA Green Master (Roche; 06 402 712 001) was used for this purpose. qPCR reactions were prepared following manufacturer´s instructions. PCR reactions were carried out in a LightCycler® 96 Instrument (Roche), programmed with 45 cycles of melting at 95 °C for 10 s, annealing at 60 °C for 10 s, and elongation at 72 °C for 10 s. The information obtained (amplification curves, melting curves and crossing points, CP, o cycle threshold, Ct) for each transcript was processed using the software provided with the LightCycler® equipment. To estimate the PCR efficiency (E), serial dilutions of the cDNA samples were amplified, and E calculated according to the equation E = 10[−1/slope] [41]. Relative quantification was based on the level of expression of a target gene relative to the level of expression of a reference gene (glyceraldehyde 3-phosphate dehydrogenase, GAPDH) [42]. Thus, for each cDNA experimental sample, the expression of a particular target gene (Table 1) relative to GAPDH expression (gene mRNA rel. GAPDH) was calculated as follows:

$${\text{Gene mRNA rel}}.{\text{ GAPDH }} = \, {{\left( {E_{{{\text{target}}}} } \right)^{{\Delta {\text{CP target}}}} } \mathord{\left/ {\vphantom {{\left( {E_{{{\text{target}}}} } \right)^{{\Delta {\text{CP target}}}} } {\left( {E_{{{\text{GAPDH}}}} } \right)^{{\Delta {\text{CP GAPDH}}}} }}} \right. \kern-\nulldelimiterspace} {\left( {E_{{{\text{GAPDH}}}} } \right)^{{\Delta {\text{CP GAPDH}}}} }}$$

**Table 1:** Sequence of primers used in qPCR

Gene name	Forward primer sequence (5′–3′)	Reverse primer sequence (5′–3′)
GAPDH	CACTGCCACTCAGAAGACTG	GGCATGTCAGATCCACAAC
FOXJ1	GACTATGCCACCAACCCACA	CGGATGGAATTCTGCCAGGT
IIIG9	ACAACCCCAGCTATGTTCGG	GGCACGTCTCGATAGAAGGG
IL-1βR1	AGAAACTCAACATACTGCCTCA	CAGCCACATTCATCACCATC
TNFαR1	TGTTGCCTCTGGTTATCTT	ACCCTCCACCTCTTTGAC

where E is the efficiency of the corresponding gene (target gene or GAPDH), ΔCP_target_ is the difference in crossing point values for the target gene obtained in the control sample and in the experimental sample, and ΔCP_GAPDH_ is the difference in crossing point values for GAPDH obtained in the control sample and in the experimental sample [42].

### Analytical methods

The statistical analysis of the data was carried out using SPSS Statistics software. The Kolmogorov–Smirnov normality tests, along with the Levene homoscedasticity test, were used to verify if data could be analysed by parametric methods. One-way or two-way analysis of variance (ANOVA) were used to compare mean values. Afterwards, the pairwise comparisons were done by the Tukey test. For the non-parametric datasets, the Kruskal–Wallis test was used, and in this case pairwise comparisons were done with Mann–Whitney *U* test. In all comparisons differences between means were considered significant when the *P* value obtained was < 0.05.

## Results

### Ependymal damage in ventricular wall explants co-cultured with NA activated microglia

**Fig. 1:**
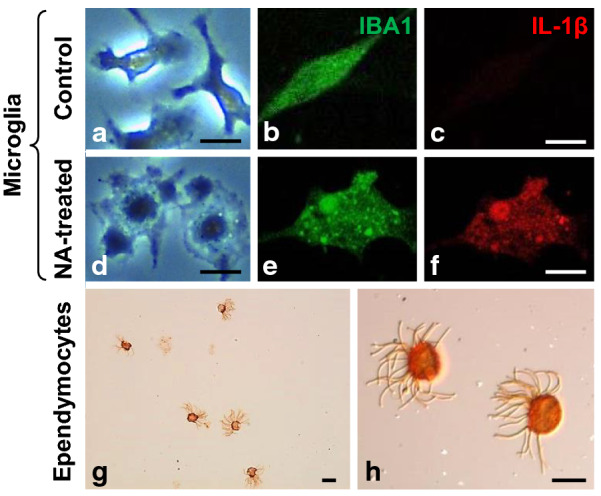
Purified microglia and ependymocyte cultures obtained for co-culture experiments. Pure microglial cultures visualized by bright field microscopy under control conditions (**a**) and after the addition of NA to the culture medium (**d**); NA induces a change in microglial morphology. Double immunofluorescence for IBA1 and the inflammatory cytokine IL-1β was performed on control (**b**, **c**) and NA-treated (**e**, **f**) microglia cultures. The identity and high purity (> 95%) of rat primary microglial cells was demonstrated by IBA1 fluorescence immunostaining (**b**, **e**). The activation of microglial cells after NA stimulation was evidenced by IL-1β labelling (**c**, **f**). Ependymal cells were likewise isolated from adult rats, and purified using an in vitro strategy. The purity (100% ependymal cells) [36] and identity of the isolated cells was evaluated by immunocytochemistry using the cilia specific marker βIV-tubulin (**g**, **h**). The bunch of cilia in ependymal cells is noticeable (**h**). Scale bars in **a**–**h**: 10 μm

Activated microglia overexpress the pro-inflammatory cytokines IL-1β and TNFα [6, 34, 43]. In a previous work by our group using pure microglial cultures obtained from mice, the addition of NA to the culture media provoked an increase in the expression, measured by qPCR, of the cytokines IL-1β, TNFα and IL-6 [40]. Here the morphology of cultured microglial cells upon NA addition was observed by bright-field microscopy (Fig. 1a, d). Double staining for IBA1 (Fig. 1b, e) and IL-1β (Fig. 1c, f) revealed undetectable levels of IL-1β in surveillant microglia (Fig. 1c). When NA was added to the culture media microglia stained with anti-IL-1β (Fig. 1f), thus confirming that NA is able to induce the expression of IL-1β in cultured microglial cells. Isolated ependymal cells were used for co-culture experiments 24 h after its purification. Under the microscope they showed cilia beating, and by inmmunocytochemistry they were βIV-tubulin positive (Fig. 1g, h). No other cell types were found in these isolates, so their purity was of 100%.

**Fig. 2:**
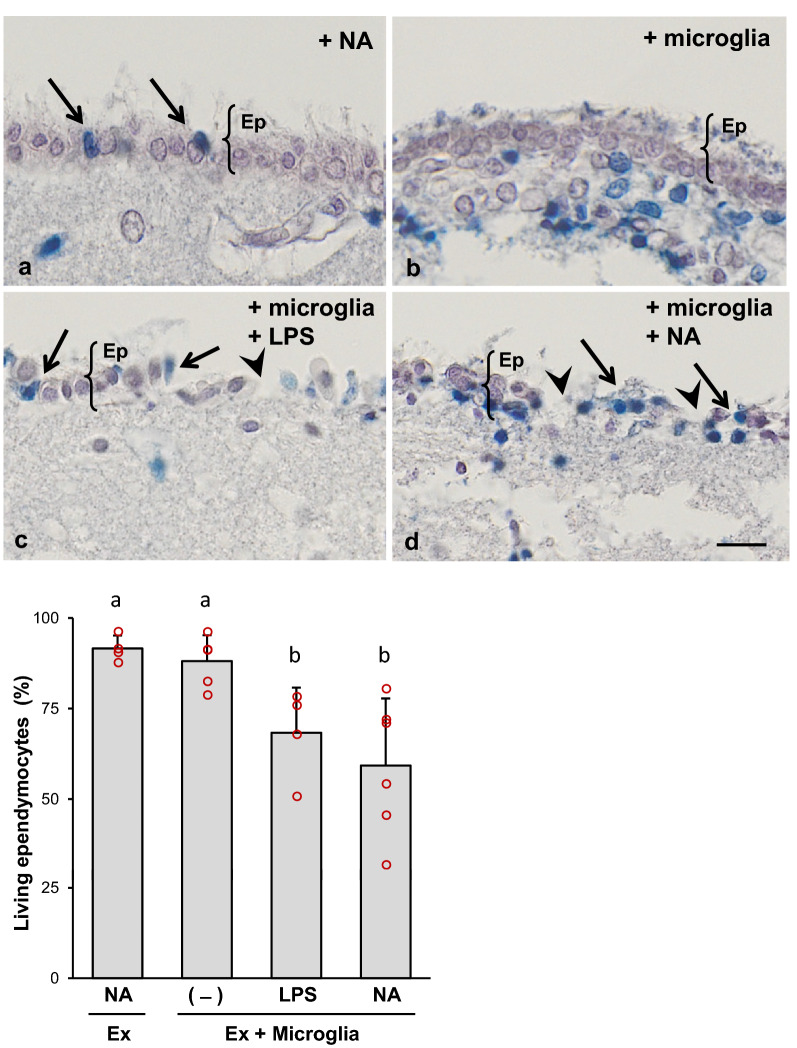
Viability of ependymocytes in ventricular wall explants co-cultured with NA-activated microglia. Septal and striatal explants with an intact ependymal cell layer were obtained from the lateral ventricles of adult rats. The explants were exposed to microglial cells, either resting (**b**) or stimulated with LPS (**c**) or NA (**d**). Some explants were exposed to NA in the absence of microglia (**a**). After 24 h, explants were stained with trypan blue, fixed, paraffin-embedded and sectioned. Dead ependymal cells were stained blue (arrows in **a**, **c** and **d**), and were easily distinguishable from alive cells, which appeared purple due to haematoxylin staining. Live and dead ependymal cells were counted, and viability was expressed as the percentage of living cells (**e**). In explants cultured alone and treated with NA (**a**) and in those co-cultured with non-stimulated microglia (**b**), only few dead ependymal cells could be found (arrows); ependymal cell viability was similar in both conditions (**e**). However, in those explants co-cultured with microglia activated either with LPS (**c**) or with NA (**d**) the ependymal layer appeared partially disrupted, with more dead cells (arrows in **c** and **d**) and some nude spaces probably due to detached cells (arrowheads in **c** and **d**). The co-culture of the explants with microglia activated with NA or with LPS provoked a similar decrease of ependymal cells viability, compared to the viability in explants only exposed to NA or cultured with non-stimulated microglia (**e**). Bars in histogram represent mean ± s.d. of *n* = 4–6 independent experiments with explants and microglial cultures obtained from different rats. Means were compared by one-way ANOVA (*P* = 0.007). Letters **a** and **b** on top of the bars indicate the absence (if the same letter appears) or existence (if different letters appear) of a significant difference between the compared conditions, as indicated by Mann–Whitney test (*P* < 0.005). Ex: explant; NA: neuraminidase added to medium; LPS: lipopolysaccharide added to medium; (-): control medium. Scale bar: 25 μm

In order to determine the effect of activated microglia on ependymal cells, we performed co-culture experiments of ventricular wall explants with pure microglial cultures. Microglia was activated by addition of NA (or LPS as positive control) to the culture medium. The percentage of living ependymocytes within the ependymal layer of the explants was evaluated 24 h after NA or LPS addition by trypan blue exclusion. In the absence of microglial cells, explants treated with NA showed a low number of dead cells (arrows in Fig. 2a). A similar result was obtained in explants that were co-cultured with non-stimulated microglia (Fig. 2b). However, the number of dead ependymal cells increased in co-cultures treated either with NA (Fig. 2d) or LPS (Fig. 2c). Not only dead cells (arrows in Fig. 2c, d) but also detached ependymal cells (arrowheads in Fig. 2c, d) could be observed. Even though sporadic dead ependymal cells (probably as a result of explant manipulation) could be found in control explants (arrows in Fig. 2a), these events significantly increased in the presence of activated microglia (arrows in Fig. 2c, d). Trypan blue positive cells were sometimes observed underneath the ependymal layer (Fig. 2b). These may correspond to other cell types which, in the context of a cultured explant, are more labile than ependymal cells, in particular those explants obtained from the striatal wall which bears the subventricular neurogenic niche.

Live/dead cell counts in several explants (*n* = 4–6) were expressed as percentage of living ependymocytes (Fig. 2e). A Kruskal–Wallis test showed that there was a statistically significant difference in ependymocytes viability between the different experimental conditions (*H* = 12.54; *P* = 0.006). Mann–Whitney multiple comparisons test revealed the lack of a significant difference in ependymal cell viability in explants treated with NA in the absence of microglia (92 ± 4%) compared to those co-cultured with non-stimulated microglia (88 ± 7%). However, in explants co-cultured with microglia, the addition of either LPS or NA induced a similar decrease in the percentage of living ependymocytes (68 ± 15% and 59 ± 19% respectively). These results demonstrate that NA-activated microglial cells compromise ependymal cells viability within the ependymal epithelium of explants.

### NA-activated microglia impair the viability of isolated cultured ependymal cells

Although the results obtained with explants indicated a role of activated microglia in ependymocytes death, the presence of other cell types in the explants might interfere in these results. Thus, an ependymal cell isolation and purification method [36] was used to perform similar co-culture experiments. An independent isolation of primary ependymocytes was carried out for each experimental replicate. For co-culture experiments, microglial cells were attached to the bottom of the multi-well plate, while ependymal cells were subsequently added and remained in suspension. Immunocytochemistry with βIV-tubulin, a specific ependymal cell marker, confirmed the purity (100%) of ependymal cells (Fig. 1g, h).

**Fig. 3:**
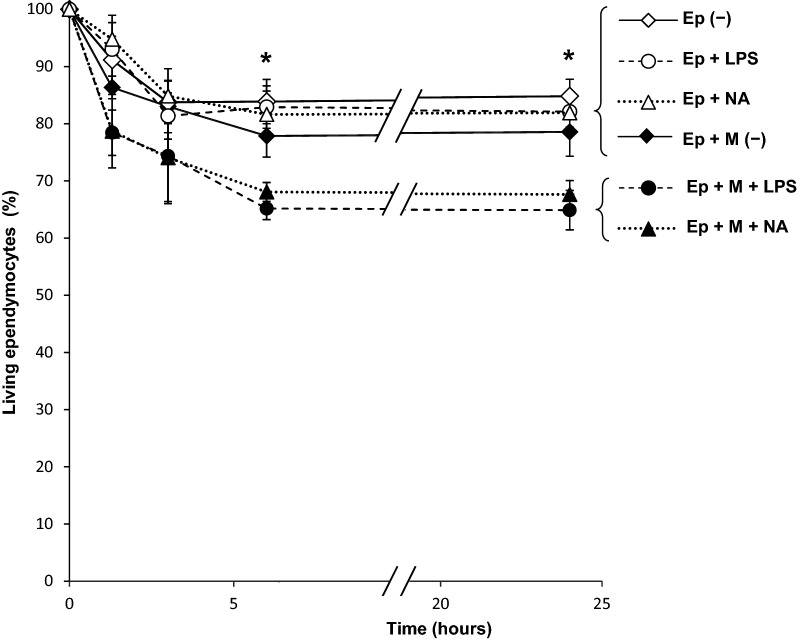
Viability of isolated ependymal cells co-cultured with microglial cells. Ependymal cell cultures were co-cultivated with resting or stimulated (either with LPS or with NA) pure microglial cells. Viability of ependymocytes was assessed at different time points, up to 24 h, by trypan blue dye exclusion. Each point represents the percentage of living ependymocytes relative to the viability measured at the beginning of the experiment (100% viability at time 0). Data are the mean ± s.d. of *n* = 6 independent experiments, where ependymal and microglial cultures were obtained from different rats. Means of viability under different culture conditions were compared by two-way ANOVA, which showed significant differences between experimental conditions (*P* < 0.001) and between time points (*P* < 0.001). The Tukey test post hoc pointed out that, after 6 h of co-culture, the viability of ependymocytes co-cultured with microglia activated either with NA or with LPS was reduced (bottom bracket), compared to the viability measured in any of the other culture conditions used as controls (top bracket), which included ependymal cells alone, with LPS or with NA, or with not-activated microglia; this difference remained at 24 h. No differences in the viability of ependymocytes co-cultured with microglia were found when using LPS or NA to activate microglia (bottom bracket). Similarly, no differences were found between control groups (top bracket). Ep: ependymal cells, M: co-culture with microglial cells; (-): control medium; LPS: lipopolysaccharide added to medium; NA: neuraminidase added to medium. * = *P* < 0.001

Pure ependymal cells were co-cultured with activated microglia, and ependymal cell viability was determined by trypan blue exclusion. The percentage of living ependymocytes was calculated at different time points up to 24 h. Negative control conditions consisted in ependymal cells cultured in the absence of microglia, or with no microglial activator (LPS or NA). Two-way ANOVA was used to compare the viability obtained under different experimental conditions. Comparisons were performed between treatments, as well as between different time points (Experimental condition: *F* = 49.9, *df* = 5, *P* < 0.001; Time: *F* = 35.8, *df* = 3, *P* < 0.001; Interaction: *F* = 1.2, *df* = 15, *P* = 0.277). This analysis revealed significant differences between experimental conditions as well as between time points, which were further examined by Tukey post hoc test (Fig. 3). Six hours after LPS or NA stimulation, the viability of ependymocytes similarly decreased to about 65–70%, but only in the presence of activated microglia (Fig. 3); no further decrease occurred up to 24 h in culture. In the absence of microglia, as well as with non-stimulated microglia, viability decreased over time, but less than in co-cultures treated with NA or LPS (*P* < 0.001). Thus, in all experimental conditions ependymocytes viability decreased up to 80–85% during the first 6 h in culture, and remained unchanged thereafter, indicating a somewhat basal impairment in the viability of ependymocytes in culture. But when ependymocytes were co-cultured with activated microglia, a larger statistically significant decrease of their viability occurred. According to our results, NA or LPS activated microglia provoke an additional 15% decrease in ependymocytes viability, thus suggesting that activated microglia jeopardize (although apparently do not completely abrogate) ependymal cell survival.

Upon microglial activation, ependymal cell death occurred relatively fast (within the first 6 h), which could be partly due to the fact that cells are in suspension and thus are more sensitive to stressors. Although we have not investigated the type of cell death, the similar pattern of trypan blue staining in dead ependymocytes either isolated or in explants may suggest a similar type of cell death. Previous observations from in vivo experiments point that, after NA injection, ependymocytes may undergo necrosis rather than apoptosis, because apoptotic bodies were not seen.

### Gene expression of IL-1β and TNFα receptors in ependymocytes

After demonstrating that activated microglia impair ependymal cells viability, we hypothesized that this effect might be mediated by inflammatory cytokines released by activated microglia, such as IL-1β [44] and TNFα [45]. First, the expression of the receptors for these cytokines was analysed in purified ependymal cells and ventricular wall explants.

**Fig. 4:**
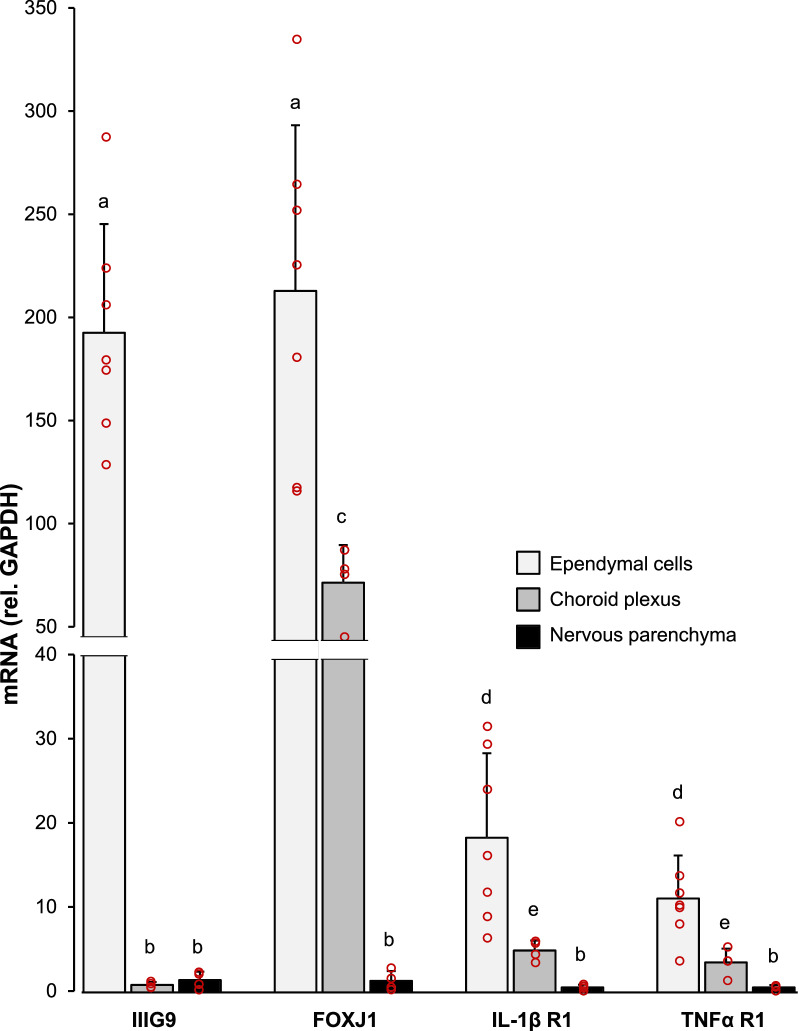
Cytokine receptors expression by isolated ependymal cells. Gene expression for the cytokine receptors IL-1β (IL-1βR1) and TNFα (TNFαR1) was quantified in pure ependymocytes by qPCR, relative to the expression of the housekeeping gene GAPDH. The expression of genes specific of ciliated cells (FOXJ1 and IIIG9) was also measured to confirm the identity of ependymocytes isolates. As a reference to compare with, the expression of the same genes was measured as well in choroid plexus (obtained from the lateral ventricles) and in brain parenchyma. Bars are the mean + s.d. of *n* = 4–7 cell isolates/tissues obtained from different rats. The high expression of IIIG9 and FOXJ1 in ependymal cells compared to the brain parenchyma indicates the enrichment of these samples in this cell type. Choroid plexus cells shared with ependymocytes the high expression of the FOXJ1, but not that of IIIG9, which was exclusively expressed by ependymal cells. The expression of the cytokine receptors IL-1βR1 and TNFαR1 was significantly higher in ependymal cells and choroid plexus, compared with the expression in the brain parenchyma. Ependymal cells expressed both receptors at a higher level than choroid plexus cells. Kruskal–Wallis test revealed differences (*P* < 0.005) between the means of the groups; pairwise comparisons were then carried out with Mann–Whitney *U* test. Letters a, b, c, d and e above bars indicate the absence (if the same letter appears) or presence (if different letters appear) of a statistical difference (*P* < 0.05). Data points have been omitted in those bars that are extremely small

The presence of the IL-1β receptor 1 (IL-1βR1) and the TNFα receptor 1 (TNFαR1) in pure ependymocytes isolated from adult rats was investigated by qPCR. Choroid plexus and brain parenchyma were used as references for comparison. Kruskal–Wallis test revealed differences (*H* = 57.7; *P* < 0.001) in the expression level of the target genes between the cells/tissues compared. The mRNA levels of ependymal cell specific genes, such as FOXJ1 and IIIG9 [46], were much higher in RNA obtained from pure ependymal cells than in choroid plexus or brain parenchyma, confirming the enrichment of ependymal isolates (Fig. 4). In choroid plexus, FOXJ1 expression was also prominent while IIIG9 was not expressed. Regarding cytokine receptors, the expression level of IL-1βR1 (*P* < 0.05) and TNFαR1 (*P* < 0.02) in ependymocytes was significantly higher than that in choroid plexus. A higher expression of both receptors was found also in choroid plexus compared to brain parenchyma, where expression levels were the lowest (Fig. 4). Thus, these results confirm the constitutive expression of IL-1βR1 and TNFαR1 in ependymal cells, at least at the mRNA level, revealing them as possible targets of the corresponding cytokines.

**Fig. 5:**
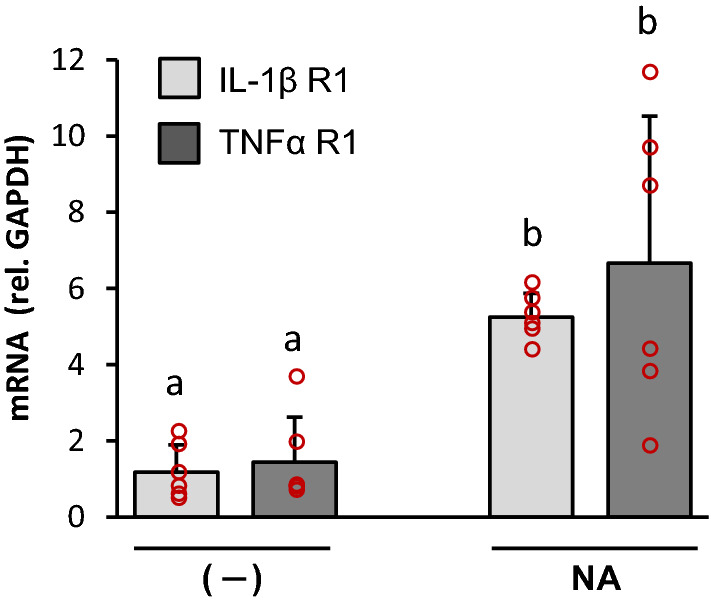
Increased expression of cytokine receptors in ventricular wall explants after stimulation with NA. The expression of two cytokine receptors, IL-1βR1 and TNFαR1, was evaluated by qPCR in control and NA-treated rat brain explants obtained from the wall of the lateral ventricles of adult rats. The mRNA levels were expressed relative to GAPDH mRNA. Bars are the mean + s.d. of *n* = 6 independent experiments with explants obtained from different rats. Kruskal–Wallis test showed significant differences (*P* < 0.001); both receptors were up-regulated after NA treatment. Letters a and b above bars indicate the absence (if the same letter appears) or presence (if different letters appear) of a statistical difference, analysed by Mann–Whitney pairwise comparisons. (*P* < 0.01)

On the other hand, the expression level of both cytokine receptors was measured in ventricular wall explants in vitro stimulated with NA for 6 h. A Kruskal–Wallis test showed statistically significant differences (*H* = 15.45; *P* < 0.002) in receptors expression between explants treated with NA and the untreated controls. Explants treated with NA showed increased expression of IL-1βR1 (*P* = 0.005) and TNFαR1 (*P* = 0.013) compared to control non-treated explants, thus indicating that NA is also able to induce the overexpression of specific cytokine receptors (Fig. 5).

### A blocking antibody against IL-1β prevents NA-induced ependymal cell death

**Fig. 6:**
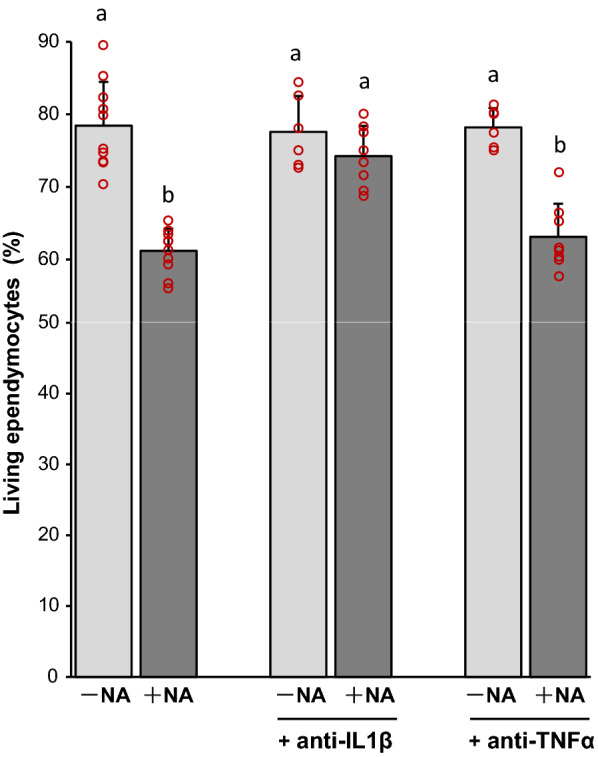
Effect of IL-1β and TNFα blocking antibodies on the viability of ependymal cells co-cultured with NA-activated microglia. Pure ependymal cells were co-cultured with rat primary microglia, which were then activated with NA. In some cultures, blocking antibodies for specific cytokines (anti-IL-1β and anti-TNFα) were added. After 24 h, the viability of ependymocytes was assessed by trypan blue dye exclusion, and the percentage of living ependymocytes calculated. Each point represents the mean + s.d. of *n* = 6–10 independent experiments, with ependymal and microglial cultures obtained from different rats. Two-way ANOVA showed significant differences between means (*P* < 0.001). Later pairwise multiple comparisons carried out by Tukey test pointed out that, as expected, the percentage of living ependymal cells decreased in co-cultures exposed to NA. However, such decrease was prevented by the addition of anti-IL-1β while, on the contrary, was not affected by the addition of anti-TNFα. Letters a and b above bars indicate the absence (if the same letter appears) or presence (if different letters appear) of a statistical difference (*P* < 0.001)

In order to investigate a potential role of the inflammatory cytokines IL-1β and TNFα in the death of ependymal cells, the microglia-ependymocytes co-culture setting was again employed, adding in this case to the culture media two different functional blocking antibodies, against IL-1β and TNFα. The viability of ependymal cells was evaluated by trypan blue exclusion (Fig. 6). Two-way ANOVA showed significant differences between ependymocytes viability according to the activation by NA (*F* = 75.09, *df* = 1, *P* < 0.001), and the presence of blocking antibodies (*F* = 8.19, *df* = 2, *P* < 0.005), finding a significant interaction of both factors (*F* = 10,29, *df* = 41, *P* < 0.001). Tukey post hoc test showed that the viability of ependymal cells significantly decreased when NA was added to the co-culture (*P* < 0.001). A similar decrease was obtained when TNFα blocking antibody was added to the media (*P* < 0.001). Conversely, the addition of IL-1β blocking antibody prevented the decrease in viability provoked by NA. Hence, these results suggest that, upon NA-induced microglial activation, IL-1β would be mediating a damage to ependymal cells that compromises their viability. Moreover, our results point that TNFα would not have such adverse effect on ependymocytes.

## Discussion

It is well documented that a single intracerebroventricular (ICV) injection of NA provokes denudation and death of the ependymal epithelium, which is not restored over time [7, 30]. In the most severe cases, that is when high NA doses are injected, the ependymal loss is complete, and an obstructive hydrocephalus develops [7]. A lower dose of NA (the model used here) results in: (i) partial ependymal loss (53.5 ± 8.5%)[29], (ii) strong microglial activation, and (iii) infiltration of leukocytes into the meninges, the cerebrospinal fluid and the brain parenchyma. Thus, this paradigm represents a model of acute aseptic neuroinflammation [6].

A unique feature of this model is the damage to the ependymal epithelium. Among the various possible causes, the contribution of the complement system to ependymal damage and death has been demonstrated, as well as the direct impact of NA itself, which may also provoke mild ependymal damage without the aid of the complement [29]. However, these causes do not fully account for all ependymal death. Thus, additional mechanisms, which are still not completely clarified, may underlie ependymal death.

Here, attention focused on microglial cells as players in ependymal damage, as they become activated upon exposure to NA. In fact, the direct activation of microglia by NA acting through the receptor TLR4 has been recently reported [40], as well as the resulting induction of inflammatory cytokines such as IL-1β, IL-6 and TNFα [40]. These cytokines could cause cellular damage, including the death of ependymal cells. The results obtained here provide evidence for this possibility. As (1) the ependymocytes isolates used here were 100% pure, (2) the expression of the receptors was detected by a quite sensitive technique such as qPCR, and (3) receptors expression was differentially detected in RNA extracts from other tissues (choroid plexus, brain parenchyma), it is very probable that ependymocytes constitutively express receptors for both IL-1β and TNFα cytokines. Furthermore, NA provoked an increase in the expression of IL-1β and TNFα receptors in ventricular wall explants, suggesting that this tissue becomes more susceptible to these inflammatory cytokines. However, demonstrating the protein expression of both receptors would be necessary to definitely confirm this finding. In this regard, the presence of IL-1βR1 in the ependyma in vivo has been previously described by other authors by both in situ hybridization and immunohistochemistry [47, 48], confirming that both the mRNA and the protein for this receptor are expressed in ependymocytes. Regarding TNFα receptor, Nadeau and Rivest [49] showed the mRNA expression in ependyma and choroid plexus by in situ hybridization, in accordance to our results. However, in a study using immunohistochemistry, TNFα receptor was not found in rat brain ependyma [50]. Furthermore, personal communication of unpublished results confirms such observation, as ependymal cells and choroid plexus appeared negative in mouse brain immunohistochemistry for TNFα receptor. Therefore, our results along with other reports indicate that ependymal cells express the receptor for IL-1β. However, and even though TNFα receptor mRNA has been detected in ependymal cells by qPCR and in situ hybridization, these cells do not seem to express a functional receptor for TNFα.

The presence of cytokine receptors in ependymal cells allows us to speculate the possibility that cytokines may mediate ependymal death. In previous works functional blocking antibodies have been used to unravel the participation of cytokines in inflammation [51, 52] and in brain cell death [53]. This strategy was used here to investigate the possible involvement of IL-1β and TNFα in ependymocyte viability. Our results strongly suggest that IL-1β impairs ependymal cell viability, whereas TNFα does not. This is in agreement with the fact that ependymal cells express IL-1β receptor but not TNFα receptors, as discussed above. Increased cell survival in hippocampus when using neutralizing IL-1β antibodies has also been described in cases of transient global ischemia [54]. Besides, the systemic infusion of IL-1β neutralizing antibodies reduced short-term brain injury after cerebral ischemia in the ovine foetus [55]. However, with these results, we cannot rule out the participation of other molecules produced by microglia (or other cells) in the death of ependymal cells.

Although the main role of microglia in the brain is protective and homeostatic, activated microglia release inflammatory cytokines that can sometimes generate neurotoxic effects [56, 57]. This fact has been described in neurons [58, 59], in Purkinje cells [60], in oligodendrocytes [61, 62] and in retinal ganglion cells [63]. Furthermore, these harmful effects of inflammatory cytokines have also been observed in experimental neuroinflammatory processes caused by inoculation of the influenza A virus, which has NA in its lipid envelope [64, 65]. This evidence supports the results obtained in the present work, that suggest the involvement of IL-1β in ependymal cell death.

Ependymal cells form a continuous epithelial layer that covers the brain ventricles and the central canal of the spinal cord [3, 8]. The integrity of the ependymal epithelium is essential for the stability of the brain ventricular system [7, 9, 32, 66]. It is well established that the loss of the ependymal cells leads to stenosis and obliteration of the cerebral aqueduct, giving rise to hydrocephalus [7, 33]. Moreover, even a dysfunction of the ciliary beating in ependymal cells generates hydrocephalus [1, 11–15]. Numerous cases of hydrocephalus with ependymal loss have been described in infections with NA-bearing viruses such as influenza, mumps, and measles viruses. [19, 20, 23–25, 27, 28]. In the model of NA-induced inflammation used here, several events concur that contribute to ependymal damage and loss: (i) activation of the complement system, with deposition of the membrane attack complex onto the apical ependymal membrane [29]; (ii) microglial cell activation and IL-1β production [34]. In vitro experiments showed that even NA by itself can cause some ependymal damage [29]. Therefore, the ependymal cell death reported in infections by NA-bearing viruses could be ascribed to the activity of NA. Despite this evidence, we cannot rule out that the behaviour of ependymal cells in vitro could be different from that in vivo. However, in vivo data support the effect of activated microglia on the viability of ependymal cells. Thus, in mice injected ICV with LPS, where an important microglial activation occurs, the death of ependymal cells was observed [67]. Also, in a similar model which uses ICV injected NA instead, there is a remarkable death of ependymal cells in vivo [6, 7, 30]. Hence, these in vivo experiments are in accordance with the in vitro results shown here and support the involvement of activated microglia in ependymal cell death.

## Conclusions

The present work presents evidence that ependymal cells viability is impaired in the presence of activated microglia, and that IL-1β released by these cells may mediate this effect. Thus, this reveals another mechanism for the ependymal damage that occurs after the ICV injection of NA, which additionally contributes to the already described deleterious actions of the complement system and NA itself. Given the relevance of ependymal cells for the integrity of the ventricular system and the susceptibility of the CNS to infections with NA-bearing viruses, it is of great importance to understand the mechanism(s) by which NA damages ependymal cells. This will be valuable information for the design of therapeutic strategies (e.g. neuraminidase inhibitors) aimed at preventing pathologies such as hydrocephalus associated to some viral infections.

## Data Availability

There is no new software, databases, and application/tool available, apart from the reported in the present article.

## Abbreviations

CNS: Central nervous system
CSF: Cerebrospinal fluid
DMEM-F12: Dulbecco’s Modified Eagle Medium/Nutrient Mixture F-12
EDTA: Ethylenediaminetetraacetic acid
FBS: Foetal bovine serum
FOXJ1: Forkhead box protein J1
GAPDH: Glyceraldehyde 3-phosphate dehydrogenase
HBSS: Hank´s balanced salt solution
IBA1: Ionized calcium binding adaptor molecule 1
ICV: Intracerebroventricular
IL-1β: Interleukin 1 beta
IL-1βR1: Interleukin 1 beta receptor 1
LPS: Lipopolysaccharide
mRNA: Messenger ribonucleic acid
NA: Neuraminidase
PBS: Phosphate buffered saline
qPCR: Quantitative polymerase chain reaction
TLR4: Toll-like receptor 4
TNFα: Tumor necrosis factor-alpha
TNFαR1: Tumor necrosis factor-alpha receptor 1
